# Classification of LAMOST spectra of B-type and hot subdwarf stars using kernel support vector machine

**DOI:** 10.1038/s41598-024-66687-6

**Published:** 2024-07-22

**Authors:** Muhammad Tahir, Bu Yude, Tahir Mehmood, Saima Bashir, Yi Zhenping, Muhammad Awais

**Affiliations:** 1 School of Mathematics and Statistics, Shandong University, Weihai, 264209 Shandong China; 2 School of Natural Sciences (SNS), National University of Sciences and Technology (NUST), Islamabad, Pakistan; 3 School of Mechanical, Electrical, and Information Engineering, Shandong University, Weihai, 264209 Shandong China; 4 School of Computer Science, Minhaj University, Lahore, Punjab Pakistan

**Keywords:** Astronomy and planetary science, Mathematics and computing

## Abstract

Machine learning has emerged as a leading field in artificial intelligence, demonstrating expert-level performance in various domains. Astronomy has benefited from machine learning techniques, particularly in classifying and identifying stars based on their features. This study focuses on the spectra-based classification of 11,408 B-type and 2422 hot subdwarf stars. The study employs baseline correction using Asymmetric Least Squares (ALS) to enhance classification accuracy. It applies the Pan-Core concept to identify 500 unique patterns or ranges for both types of stars. These patterns are the foundation for creating Support Vector Machine (SVM) models, including the linear (L-SVM), polynomial (P-SVM), and radial basis (R-SVM) kernels. Parameter tuning for the SVM models is achieved through cross-validation. Evaluation of the SVM models on test data reveals that the linear kernel SVM achieves the highest accuracy (87.0%), surpassing the polynomial kernel SVM (84.1%) and radial kernel SVM (80.1%). The average calibrated accuracy falls within the range of 90–95%. These results demonstrate the potential of using spectrum-based classification to aid astronomers in improving and expanding their understanding of stars, with a specific focus on the identification of hot subdwarf stars. This study presents a valuable investigation for astronomers, as it enables the classification of stars based on their spectra, leveraging machine learning techniques to enhance their knowledge and insights in astronomy.

## Introduction

B-type stars and hot subdwarf stars are both intriguing objects in the field of astronomy. B-type stars, also identified as main-sequence B stars, represent a subset of massive, young stars currently in the hydrogen-burning phase. Positioned along the main sequence on the Hertzsprung-Russell (HR) diagram^1^, these stars exhibit distinctive features such as high temperatures, luminosities, and spectral characteristics dominated by ionized helium and metals.

Contrary to main-sequence stars of comparable mass, hot subdwarf stars constitute a distinct class characterized by heightened temperatures and luminosities. Positioned below the main sequence on the Hertzsprung-Russell diagram, their unique evolutionary path hints at specialized formation mechanisms^2^. The genesis of hot subdwarfs is likely linked to interactions with companion stars or systems, resulting in the shedding of their outer envelopes. Despite their relative rarity, the study of hot subdwarfs provides invaluable insights into stellar evolution and the intricacies of binary system formation.

The motivation for classifying B-type and hot subdwarf stars specifically lies in their distinct scientific importance and the availability of high-quality data. B-type stars are crucial for understanding early stellar evolution, while hot subdwarf stars represent advanced evolutionary stages often associated with binary interactions. By focusing on these two types, we aimed to demonstrate the efficacy of our classification method with a robust dataset, comprising 11,408 B-type and 2422 hot subdwarf stars. Introducing additional types in this initial study could complicate the model without providing clear incremental insights. Future research will extend this methodology to include more star types.

In the late 1990s, powerful telescopes ushered in a data surge in astronomy. Surveys like the Hamburg Quasar Survey^3^, Hamburg ESO Survey^4^, and Edinburgh-Cape Survey^5^ identified over 2000 hot subdwarf stars during that period.Today, modern telescopes continue this trend, providing extensive datasets. The Javalambre Photometric Local Universe Survey^6^ spans the solar system to cosmology, exploring low-metallicity stars^7^ and galaxy formation^2^.The Sloan Digital Sky Survey (SDSS)^8,9^ has been a cornerstone since 2006, contributing nearly 2000 hot subdwarf spectra and doubling available subdwarf data^10,11^.LAMOST (Large Sky Area Multi-Object Fiber Spectroscopic Telescope) in China, notably, has been pivotal in diverse astronomical investigations, including hot subdwarf star classification^12^.

Astronomy relies heavily on the acquisition, storage, and analysis of extensive datasets. Over time, various data acquisition techniques and models have been developed to enhance the field, with advanced technologies like particle detectors, telescopes, and numerical simulations revolutionizing modern data acquisition in astronomy. The past two decades have seen significant advancements in observational and computational capabilities driven by optical, LAMOST, and computer technologies. These advancements have resulted in an unprecedented influx of data, commonly referred to as Big Data, necessitating the development of new analysis methods to enable scientific discoveries^13^. The surge in data has been widely acknowledged^14–17^. Astronomers have turned to machine learning and data mining techniques to handle these massive datasets, providing powerful tools to leverage data and gain insights into astronomical objects and spectral behavior^18–21^. The combination of machine learning and data mining methodologies enables astronomers to unlock valuable information from the vast amount of available data, aiding in identifying astronomical objects and facilitating comprehensive spectra analysis.

The Large Sky Area Multi-Object Fiber Spectroscopic Telescope (LAMOST)^12^, also known as the Guo Shoujing Telescope, represents a valuable resource for expanding our understanding of hot subdwarf stars. With its tenth data release (LAMOST DR10) comprising 10,000 spectra collected from the years 2017 to 2023, LAMOST offers a targeted dataset for studying these celestial objects. In our investigation, we ultimately presented approximately 10,000 hot subdwarf candidates. From these candidates^22^, identified 139 new hot subdwarf stars, further solidifying the importance of LAMOST in expanding our catalog and understanding of these intriguing stellar objects. This focused approach allows for a more detailed analysis of hot subdwarf stars within a specific timeframe. Additionally, the ability to derive stellar parameters from LAMOST spectra opens avenues for enhancing our understanding of various astrophysical phenomena. By leveraging this dataset, we can address key issues such as the discrepancy in the predicted positions of helium subdwarf O-type stars, identifying counterparts of blue hook stars from globular clusters, elucidating abundance patterns in sdB stars to clarify diffusion processes, and uncovering binary sdB systems with massive white dwarf companions. These endeavors contribute significantly to advancing our comprehension of hot subdwarf stars and their role in stellar evolution.

Correcting the spectrum baseline is crucial, involving subtracting the absorbance value at a specific wavelength from all wavelengths in the sample spectrum. This correction is necessary to mitigate instrument noise and the impact of light-scattering particulates in the sample, which may introduce an offset in the overall absorbance^23,24^. Over time, several modified methods, inspired by Asymmetric Least Squares (ALS), have been developed to enhance baseline correction accuracy. These modifications include adaptive iteratively reweighted penalized least squares^25^, improved asymmetric least squares^26^, asymmetrically reweighted penalized least squares^27^, adaptive smoothness parameter penalized least squares^28^, and a modified penalized least squares smoothing technique^29^. These advancements aim to elevate the quality of spectral analysis by effectively addressing challenges posed by instrument noise and the presence of interfering particulates.

In this study, we applied the Asymmetric Least Squares (ALS) method for baseline correction, following the approach proposed by^23^. To classify stars based on spectral data, we employed the support vector machine (SVM) technique^30^ with various kernels, including the linear kernel (L-SVM)^31^, polynomial kernel (P-SVM)^32^, and radial basis kernel (R-SVM)^33^. The SVM constructs a hyperplane to maximize the separation between different classes, with classification accuracy serving as a metric to evaluate the model’s predictive performance. Looking ahead, there is a demand for innovative approaches to classify extensive astronomy databases and identify multiple stars. These advancements are expected to further enhance the accuracy and efficiency of star classification techniques.

The remainder of the paper is organized as follows: Section “Methods” discusses the data set. Section “Results and discussion” presents the pan core k-means clustering-based method and the classification method. The results are presented in Section ”Conclusions”.

### Data acquisition

The Large Sky Area Multi-Object Fiber Spectroscopic Telescope (LAMOST), situated at the Xinglong Observatory in Northern China, stands as one of the world’s largest telescopes^12^. Boasting a primary mirror diameter of 4 meters, LAMOST was specifically designed for a comprehensive spectroscopic survey, targeting millions of stars and galaxies. The telescope’s distinct multi-object spectroscopy capability allows it to simultaneously study up to 4000 objects. This is made possible by utilizing optical fibers to guide light from each object to spectrographs, enabling the analysis of various properties such as chemical composition and velocity. Notably, LAMOST features a Schmidt mirror telescope^4^ with a wide field of view exceeding 3000 square degrees in a single observation-more than seven times the size of the full moon. Since its inauguration in 2008, LAMOST has successfully executed several survey phases, including the LAMOST Pilot Survey^34^ and the LAMOST Experiment for Galactic Understanding and Exploration (LEGUE)^35^. These surveys have yielded extensive and valuable data, contributing significantly to studies on diverse astrophysical topics, including Galactic structure, stellar kinematics, and the historical chemical enrichment of the Milky Way.

Our research draws upon data from two pivotal resources: the LAMOST Data Release 8 (DR8) and the LAMOST Data Release 10 (DR10) catalogs, crucial repositories of stellar spectra.The spectra in LAMOST DR8 and LAMOST DR10 exhibit notable differences. Firstly, both DR8 and DR10 spectra benefit from enhanced flux calibration, improving the accuracy and reliability of the data compared to previous releases. Secondly, all classifications in DR8 were done automatically, ensuring consistency across classifications, while DR10 was utilized primarily for searching for new hot subdwarf candidates. However, the most significant distinction lies in the increased diversity of stellar types present in DR10. While this expansion broadens the scope of our analysis, it also introduces complexities, making it more challenging to identify hot subdwarf stars amidst a multitude of spectra. Notably, DR10 encompasses a higher proportion of unknown objects-objects that defy classification into known categories-further complicating the classification task. These unknown objects, considered noise in our model, may arise from various sources such as faulty camera pixels, cosmic-ray hits, or rare objects undetectable by the LAMOST data processing pipeline. Consequently, the process of discovering hot subdwarf stars using DR10 data is inherently more intricate and demanding. Nevertheless, by navigating these challenges with advanced methodologies, we endeavor to unlock new insights into the nature and behavior of hot subdwarf stars, furthering our understanding of stellar astrophysics.

**Figure 1:**
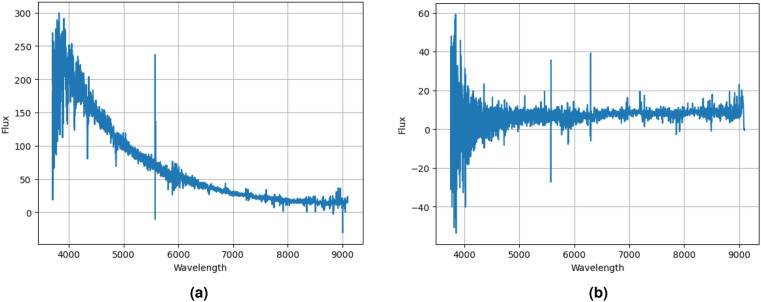
Spectra selected from the LAMOST DR8 catalog.

Figure 1a displays the spectrum of hot subdwarf stars, and Fig. 1b displays the spectrum of B-type stars specifically from the LAMOST DR8 dataset. These spectra provide valuable information about the light intensity emitted by these hot subdwarfs. The LAMOST DR8 catalog provided a rich stellar spectra dataset, allowing us to explore and classify 11408 B-type and 2422 hot subdwarf stars. The spectra were obtained using the LAMOST (Large Sky Area Multi-Object Fiber Spectroscopic Telescope) instrument, which covers a wide spectral range with moderate resolution. We selected a subset of spectra from the catalog, focusing on 11408 B-type stars and 2422 hot subdwarf stars based on known spectral characteristics and classification criteria. These spectra were preprocessed to extract relevant features for subsequent classification using a kernel support vector machine (SVM) algorithm.

To identify potential hot subdwarf candidates, this study utilized the LAMOST DR10 catalog and implemented a binary classification approach. The aim is to efficiently and accurately separate the first class from the second class. This task is a binary classification problem, where our model will assign the label 1 or 2 to each object, and the objects labeled 1 will be considered as hot subdwarf star candidates. These stars are obtained by cross-matching the LAMOST DR10 catalog with the catalog of known hot subdwarf^36^.

The data acquisition process involved capturing spectra within a fixed wavelength range using the difference method and aligning flux to a consistent wavelength through interpolation. The first 4096 columns of the dataset represented flux within the wavelength range of 3800-9100.

To ensure consistency and comparability, the data underwent a preprocessing step, ensuring that the subsequent analyses were conducted on a standardized and refined dataset.

## Methods

The classification of B-type and hot subdwarf stars presents several technical challenges. Firstly, the spectra of these stars can have overlapping features, making accurate differentiation difficult. Effective baseline correction is crucial; therefore, we used Asymmetric Least Squares (ALS) to remove noise and enhance signal quality. Identifying the most relevant features from the spectra is another significant challenge. We addressed this by employing the Pan-Core concept to identify 500 unique patterns essential for classification.

Traditional machine learning methods have been explored extensively. In this paper, the Pan-Core concept based on K-means for training data acquisition, and Support Vector Machine (SVM) for classification of star data is implemented. The Pan-Core concept utilizes K-means to identify and select representative samples from the available training data, aiming to construct a robust classification model.

Model selection and parameter tuning significantly affect the performance of the classification. We evaluated three SVM kernels (linear, polynomial, radial basis) and used cross-validation for optimal tuning. Furthermore, the research examines the effect of different kernel functions within SVM on the accuracy and performance of star classification. The choice of the kernel function plays a crucial role in capturing and separating the underlying patterns in the data.

SVMs offer several advantages over other methods such as decision trees, ensemble learning, and neural networks/deep learning for this spectral classification problem. SVMs are less prone to overfitting compared to decision trees and perform well in high-dimensional spaces, which is crucial for spectral data. They maximize the margin between classes, aiding in the distinction between overlapping spectral features of B-type and hot subdwarf stars. The use of kernel functions allows SVMs to handle non-linear relationships effectively. Additionally, SVMs are computationally more efficient and require less data than deep learning models, with simpler model interpretation and fewer hyperparameters to tune. These characteristics make SVMs particularly well-suited for our classification task.

Additionally, the data imbalance between the more numerous B-type stars and the fewer hot subdwarf stars can bias the model. We mitigated this by ensuring balanced training through appropriate sampling techniques. The model can effectively classify new star data using SVM based on the learned patterns from the training samples.

**Figure 2:**
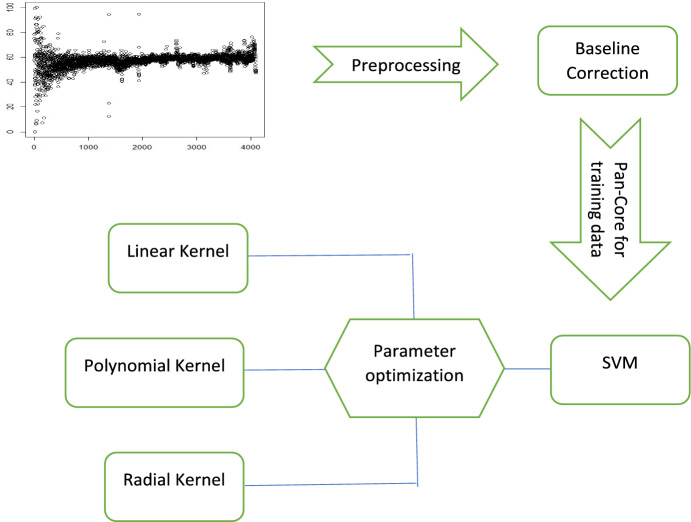
The presented figure showcases the selected methodology’s flow chart.

The authors present a flow chart in Fig. 2 to visually represent the adopted methodology. This flow chart outlines the sequential steps and procedures involved in acquiring the training data, training the model using K-means and SVM, and ultimately classifying the star data. By implementing this approach and analyzing the impact of the kernel function, the study aims to enhance the accuracy and efficiency of star classification, contributing to a deeper understanding of celestial objects and their characteristics.

### Pre-processing

In star spectroscopy, the spectra of stars are typically composed of absorption or emission lines superimposed on a continuum of emission. These spectral features arise from various physical processes occurring within the star, providing crucial information about its composition, temperature, and other fundamental properties. The deviation from the expected smoothness at zero intensity is a consequence of the presence of these absorption or emission lines and their interactions with the continuum emission.

To address these distortions and accurately interpret the spectral features, baseline correction procedures are utilized. These procedures aim to mitigate systematic variations in the intensity baseline, thereby improving the clarity of the spectral information. However, the effectiveness of baseline correction procedures depends on tuning parameters that need to be carefully selected.

In this study, instead of relying on subjective approaches, we adopted an objective procedure for choosing the baseline correction method^37^. This method outlines an optimal and systematic approach to selecting the most suitable baseline correction technique for the given star spectroscopy data.

By employing this objective procedure, our goal is to eliminate potential biases and ensure the selection of a baseline correction method that aligns best with the specific characteristics of the star spectra being analyzed. This objective approach enhances the reliability and reproducibility of the baseline correction process, leading to a more accurate and meaningful interpretation of the star spectroscopic data. It also provides a standardized methodology that can be applied consistently across different datasets, improving the overall quality and comparability of the results obtained.

The algorithm states

1. For each baseline correction algorithm, determine the appropriate levels at which all parameters will be tested.
2. Using the corresponding algorithm, the baseline is corrected at each parameter level.
3. Utilize the corrected baseline spectral data to model responses related to the physical characteristics of the stars, employing Partial Least Squares (PLS) regression.
4. Validate the model’s prediction capability to assess its accuracy in forecasting the relevant spectral features.
5. The optimal levels of parameters are determined for each baseline correction algorithm.
6. The baseline correction algorithm with the best prediction capability is selected as the optimal choice among all the algorithms considered.

The evaluation of prediction capability typically involves assessing cross-validated accuracy. This process includes performing cross-validation, where the data is divided into subsets for both training and testing the model. This division allows for an estimation of the model’s predictive performance.

The potential Asymmetric Least Squares (ALS) method is briefly explained below.

### Asymmetric least squares (ALS)

The Asymmetric Least Squares (ALS)^23,24^ method is a powerful approach used for data analysis utilizes the least square method to effectively handle predictor variables with significant errors. By assigning appropriate weights, the ALS method downplays the influence of variables with substantial errors while considering their impact on the analysis.

To achieve a smooth and accurate representation of the data, the ALS method incorporates 2nd derivative restriction within its smoothing process. This constraint helps balance the trade-off between achieving smoothness and preserving the relevant features present in the dataset.

The ALS method is mathematically expressed as:

1 $$\begin{aligned} S = \sum w_i (x_i - b_i)^2 + \lambda \sum (\Delta ^2 b_i) \ \end{aligned}$$

Here, $x_i$ represents the original spectrum, $b_i$ denotes the estimated baseline, $w_i$ corresponds to the asymmetric residual weights, and $\Delta ^2$ represents the second derivative of the estimated baseline. ALS aims to minimize the value of the expression $S$ by adjusting the baseline estimates.

To fine-tune the ALS algorithm, there are two adjustable parameters: - $\lambda$ is the smoothing parameter, which controls the degree of smoothness applied to the estimated baseline. - $w$ represents the weight assigned to the asymmetric residual, allowing for flexibility in handling different degrees of errors in predictor variables.

By appropriately adjusting these parameters, we customize the behavior of the ALS method according to the specific characteristics of our data. This flexibility enhances the ALS algorithm’s adaptability and improves its performance in accurately estimating baselines and revealing meaningful patterns in various analytical scenarios.

### Pan-core spectrum training data acquisition

The study involves a substantial dataset of hot subdwarf and B-type star spectra. To overcome the challenge of training a model on such a vast dataset, we employed the pan-core concept, originally developed in genomics^38^, as the basis for training data acquisition. The pan-core concept involves the following steps:

1. Utilizing K-means clustering with a large value of K within each class.
2. Employing the nearest neighborhood method to extract $s$ samples that are closest to each centroid obtained from K-means clustering.

Through the implementation of these steps, we curated a comprehensive set of $Ks$ samples for each class, guaranteeing the inclusion of various spectral representations within our training dataset. It’s imperative to highlight that the input for K-means clustering consisted of pre-processed flux spectra, with the input dimension explicitly defined. This meticulous approach efficiently encapsulates the intrinsic characteristics of star spectra, facilitating the model’s learning process with a manageable yet informative subset of samples.

**Figure 3:**
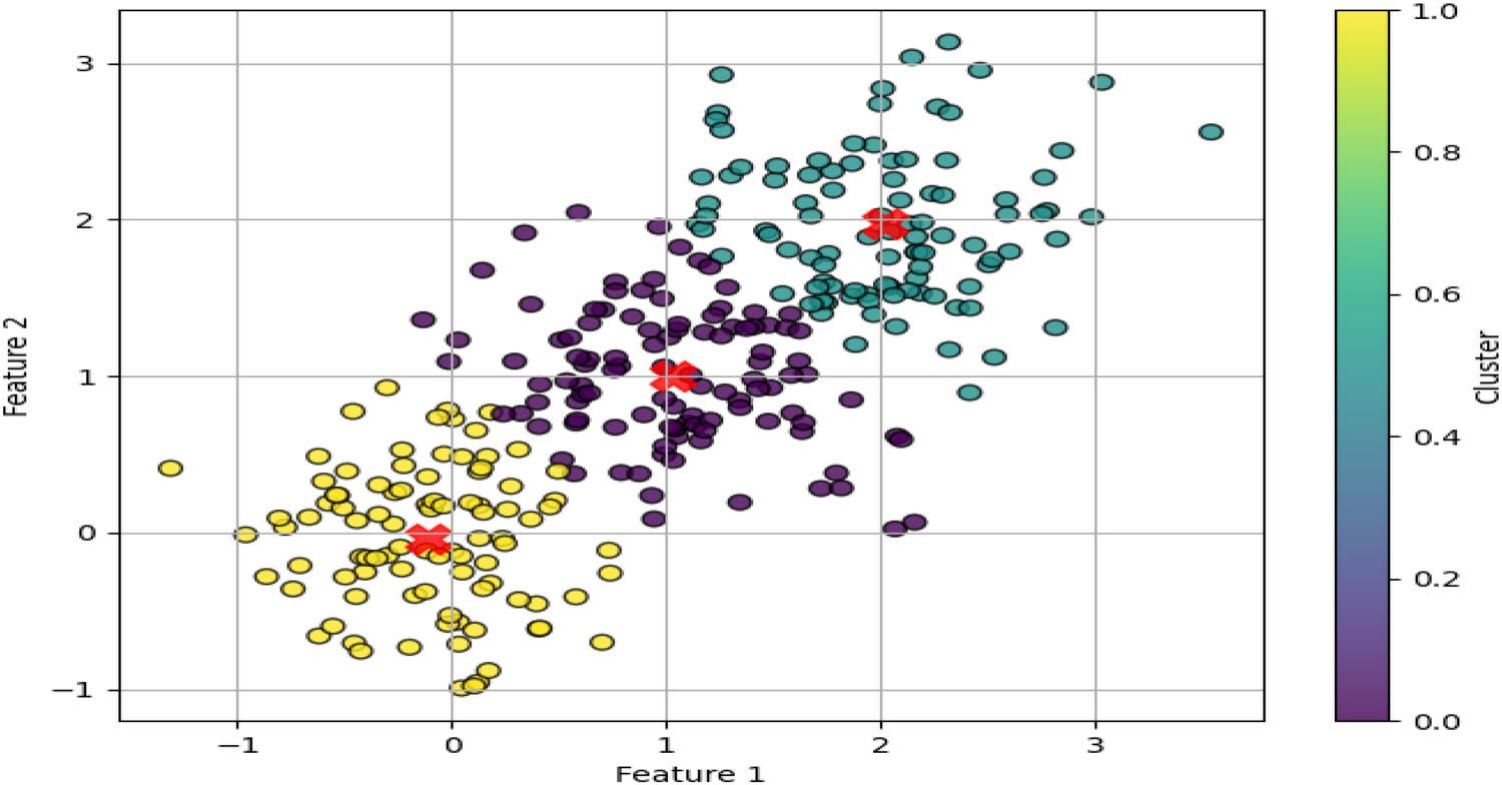
K-means clustering applied to synthetic spectral data, demonstrating the partitioning of data points into clusters represented by different colors. Red crosses denote the centroids of each cluster.

The integration of the pan-core concept in star spectra analysis significantly diminishes the dimensionality of training data while upholding pivotal features and ensuring a comprehensive portrayal of spectral diversity within each class, as depicted in Fig. 3. This enhancement empowers our model to extract insights from a discerning subset of samples, thereby amplifying its accuracy and generalization capabilities in spectral classification tasks.

### Support vector machines

Support vector machine (SVM) is a powerful supervised machine learning algorithm initially introduced by Cortes and Vapnik^30^. It is widely utilized for both classification and regression tasks due to its ability to handle various types of data through the use of different kernels. SVM offers flexibility in choosing kernels such as linear (L-SVM), polynomial (P-SVM), and radial basis (R-SVM)^31–33^, allowing for effective modeling of complex relationships within the data.

SVMs are well-suited for spectral classification problems due to several characteristics. They are effective in high-dimensional spaces, which is important given the complexity and dimensionality of spectral data. They are robust to overfitting, especially in cases where the number of features exceeds the number of samples, as often seen in spectral datasets. SVMs also perform well with clear margin separation, which helps distinguish between B-type and hot subdwarf stars with overlapping spectral features. Additionally, SVMs can utilize different kernel functions to handle non-linear relationships in the data, enhancing classification accuracy. Cross-validation for parameter tuning ensures optimal model performance, making SVMs a reliable choice for this classification task. These characteristics make SVMs particularly suited for the spectral classification of stars.

Several variables are introduced to elucidate the workings of Support Vector Machines (SVM) in classifying star spectra data. These variables include $J$, representing the total number of training samples; $x_j$ and $y_j$, denoting the features and labels of each sample, respectively; $x$ and $y$, representing the feature space and class labels, with $y$ taking values of either -1 or 1; $w$, signifying the coefficient vector; $b$, representing the bias term; $\alpha _j$, indicating the Lagrange multipliers associated with each training sample; $\theta (x)$, denoting the feature mapping function; $S(w, x)$, signifying the inner product between $w$ and $x$; and $C$, representing the regularization parameter in soft-margin SVM. Each variable plays a crucial role in the formulation and optimization of the SVM algorithm, contributing to its effectiveness in accurately classifying star spectra data.

In SVM, there is a constraint given by: $\sum _{j=1}^{J} y_j \alpha _j = 0$ $(x_j,y_j), \quad x \in R^d, y \in \{-1,1\}$ where $(x_j, y_j)$ represents the training samples, with $x$ belonging to the $d$-dimensional space and $y$ taking values of either – 1 or 1. The aim of SVM is to find a linear classifier in an infinite-dimensional space, given by:

2 $$\begin{aligned} f(x) = \text{sign}(w \cdot \theta (x) + b) \end{aligned}$$

Here, $w \cdot \theta (x) = S(w, x)$ denotes the inner product between the coefficient vector $w$ and the input sample $x$.

SVM’s strength lies in its ability to separate data points by defining a decision boundary while maximizing the margin between different classes. The choice of a kernel function determines the transformation of the input data into a higher-dimensional space, enabling effective separation of classes that may not be linearly separable in the original feature space.

By utilizing SVM with different kernels, we explore diverse strategies to classify the star spectra data effectively. The linearity of L-SVM, the flexibility of P-SVM, and the radial basis function of R-SVM provide distinct approaches for capturing the underlying patterns and relationships within the data. This versatility allows for a comprehensive analysis of the star spectra and enhances the model’s capability to make accurate classifications.

In our study, the soft-margin Support Vector Machine (SVM)^39^ formulation is essential for effectively classifying star spectra data. The key variables involved in the soft-margin SVM include $\theta ^*_\text{soft}(w)$, representing the optimized parameter; *C*, signifying the regularization parameter controlling the trade-off between achieving a smooth decision boundary and accurately classifying training data points; $\xi _j$, indicating the slack variables that allow for misclassifications in the optimization process; $L_\text{soft}(w, b, \alpha , \xi )$, denoting the soft-margin SVM objective function; and $W_\text{soft}(\alpha )$, representing the dual cost function for soft-margin SVM. By fine-tuning the *C* parameter, we aim to strike the right balance between maximizing the margin between classes and minimizing misclassifications, ensuring optimal classification performance for star spectra data analysis.

For soft-margin SVM, optimization is given by:

3 $$\begin{aligned} \theta ^*_\text{soft}(w) = \text{argmin}_{w, \xi } \frac{1}{2} \Vert w\Vert ^2 + C\sum _{j=1}^{J} \xi _j \end{aligned}$$

such that,

4 $$\begin{aligned} \ y_j (w \cdot \theta (x_j) + b) \ge 1 - \xi _j \ \end{aligned}$$

$$\begin{aligned} \xi _j \ge 0 \end{aligned}$$

5 $$\begin{aligned} \begin{array}{rl} L_\text{soft}(w, b, \alpha , \xi ) =&\frac{1}{2} \Vert w\Vert ^2 + C \sum _{j=1}^{J} \xi _j - \sum _{j=1}^{J} \alpha _j \left( y_j (w \cdot \theta (x_j) + b) - 1 + \xi _j\right) \end{array} \end{aligned}$$

The objective of the soft-margin SVM optimization is to minimize the above function, denoted by $\theta ^*_\text{soft}(w)$, with respect to the coefficients $w$ and slack variables $\xi _j$, where $C$ controls the trade-off between margin maximization and error minimization.

The stationary conditions are,

6 $$\begin{aligned} \frac{\partial L_\text{soft}}{\partial w}= & {} w - \sum _{j=1}^{J} y_j \alpha _j \theta (x_j) = 0 \end{aligned}$$

7 $$\begin{aligned} \frac{\partial L_\text{soft}}{\partial b}= & {} \sum _{j=1}^{J} y_j \alpha _j = 0 \end{aligned}$$

8 $$\begin{aligned} \frac{\partial L_\text{soft}}{\partial \xi _j}= & {} C - \alpha _j = 0 \end{aligned}$$

9 $$\begin{aligned} \alpha _j (y_j (w \cdot \theta (x_j) + b) - 1 + \xi _j)= & {} 0 \end{aligned}$$

These stationary conditions define the critical points of the Lagrangian $L_\text{soft}$, where the partial derivatives with respect to the parameters $w$, $b$, and $\xi _j$ are equated to zero.

So the weight vector is a linear combination of the data points:

10 $$\begin{aligned} w = \sum _{j=1}^{J} y_j (\alpha _j - \alpha _j^*) \theta (x_j) \end{aligned}$$

The weight vector $w$ is expressed as a linear combination of the support vectors $x_j$, weighted by the corresponding Lagrange multipliers $\alpha _j - \alpha _j^*$.

Then the classifier is:

11 $$\begin{aligned} f_\text{soft}(x)= & {} \text{sign}\left( \sum _{j=1}^{J} y_j (\alpha _j - \alpha _j^*) \theta (x_j) \cdot \theta (x) + b\right) \end{aligned}$$

12 $$\begin{aligned}= & {} \text{sign}\left( \sum _{j=1}^{J} y_j(\alpha _j - \alpha _j^*) S(x_j,x) + b\right) \end{aligned}$$

The soft-margin classifier $f_\text{soft}(x)$ is determined by the sign of the inner product of the support vectors $x_j$ with the input sample $x$, weighted by the differences in Lagrange multipliers $\alpha _j - \alpha _j^*$, and added to a bias term $b$.

Substituting into the Lagrangian gives the dual cost function for soft-margin SVM:

13 $$\begin{aligned} W_\text{soft}(\alpha ) = \sum _{j=1}^{J} \alpha _j - \frac{1}{2} \sum _{j,i} y_j y_i (\alpha _j - \alpha _j^*) (\alpha _i - \alpha _i^*) S(x_j,x_i) \end{aligned}$$

The dual cost function $W_\text{soft}$ captures the trade-off between maximizing the margin and minimizing classification errors, where $\alpha _j$ are the Lagrange multipliers associated with each support vector.

The optimization for soft-margin SVM is now:

14 $$\begin{aligned} {\hat{\alpha }}_\text{soft} = \arg \max _\alpha W_\text{soft}(\alpha ) \end{aligned}$$

such that,

15 $$\begin{aligned} 0 \le \alpha _j \le C \end{aligned}$$

The optimal Lagrange multipliers ${\hat{\alpha }}_\text{soft}$ are obtained by maximizing the dual cost function $W_\text{soft}$ subject to the constraints $0 \le \alpha _j \le C$, ensuring that the Lagrange multipliers are within a feasible range.

16 $$\begin{aligned} f_\text{soft}(x)= & {} \text{sign}\left( \sum _{j=1}^{J}y_j (\alpha _j - \alpha _j^*) S(x_j,x) + b\right) \end{aligned}$$

17 $$\begin{aligned}= & {} \text{sign}\left( \sum _{y_j:j=1} (\alpha _j - \alpha _j^*) S(x_j,x) \right. \left. - \sum _{y_i:i=-1} (\alpha _i - \alpha _i^*) S(x_i,x) + b\right) \end{aligned}$$

18 $$\begin{aligned} f_\text{soft}(x)= & {} \text{sign}\left( h_+(x) - h_-(x) + b\right) \end{aligned}$$

The final soft-margin classifier $f_\text{soft}(x)$ predicts the class label of an input sample $x$ based on the sign of the decision function $h_+(x) - h_-(x) + b$, where $h_+(x)$ and $h_-(x)$ are the contributions from positive and negative support vectors, respectively, to the decision function.

In support vector machines (SVM), the parameter $C$ plays a crucial role as the regularization parameter, influencing the balance between achieving a smooth decision boundary and accurately classifying training data points. A smaller value of $C$ promotes a broader margin, allowing for a more generalizable model but potentially compromising on fitting the training data precisely. Conversely, a larger $C$ value results in a narrower margin, potentially fitting the training data more closely but risking overfitting and reduced generalization to unseen data. Fine-tuning the $C$ parameter is essential to find the right balance for SVM, ensuring effective classification while avoiding underfitting or overfitting issues in various applications, including our star spectra data analysis.

#### Linear kernel SVM (L-SVM)

The Linear kernel is a fundamental kernel function specifically designed for dealing with linearly separable data. It allows for the transformation of data points into a higher-dimensional space to facilitate linear separation.

The mathematical formula for the Linear kernel is given by:

19 $$\begin{aligned} F(x_j) \cdot F(x_k) = (x_j \cdot x_k)^2 \end{aligned}$$

This equation represents the inner product of the transformed feature vectors $F(x_j)$ and $F(x_k)$, which is obtained by squaring the dot product of the original data points $x_j$ and $x_k$.

In a simplified form, the expression for the Linear kernel can be represented as:

20 $$\begin{aligned} F(x_j, x_k)= x_j \cdot x_k + c \end{aligned}$$

Here, $c$ represents a constant term. This formulation enables the calculation of the dot product between the input vectors $x_j$ and $x_k$, with the addition of the constant term $c$.

While the Linear kernel in Support Vector Machines (SVM) offers simplicity and computational efficiency, it is crucial to delve into its inherent characteristics for effective utilization. The Linear kernel is particularly adept at handling linearly separable data by defining a decision boundary in the original feature space. Unlike its counterparts, such as the Polynomial or Radial Basis Function (RBF) kernels, the Linear kernel doesn’t involve complex transformations into higher-dimensional spaces. This simplicity not only contributes to computational efficiency but also provides transparency in understanding the decision-making process. Additionally, the absence of kernel-specific parameters in the Linear SVM simplifies the tuning process, making it more straightforward for practitioners. Despite its simplicity, the Linear kernel remains a powerful tool, especially when dealing with large-scale datasets, where its efficiency and interpretability become advantageous in various applications.

#### Polynomial kernel SVM (P-SVM)

The Polynomial kernel is a non-stationary kernel that can be applied to both hard-margin and soft-margin classification scenarios. It is particularly well-suited for problems where all the training data has been normalized, ensuring consistency across the dataset.

The mathematical representation of the Polynomial kernel is as follows:

21 $$\begin{aligned} \ F(x_j, x_k) = (\alpha x_j \cdot x_k + c)^d \ \end{aligned}$$

In this equation, $F(x_j, x_k)$ represents the transformed feature vectors obtained by raising the dot product of the input vectors $x_j$ and $x_k$ to the power of the polynomial degree $d$. The parameters $\alpha$, $c$, and $d$ are adjustable and play significant roles in shaping the behavior and performance of the Polynomial kernel.

By adjusting these parameters, we can control the complexity and flexibility of the kernel function, allowing it to adapt to different types of data and classification problems. The parameter $\alpha$ determines the influence of the dot product term, $c$ represents a constant offset, and $d$ determines the degree of the polynomial transformation. Fine-tuning these parameters is essential to achieve optimal performance and generalization in Polynomial kernel-based SVM models.

The flexibility of the Polynomial kernel makes it a valuable tool for handling data sets with complex relationships and non-linear decision boundaries. By leveraging the adjustable parameters, researchers can effectively explore the trade-off between model complexity and generalization, ensuring that the Polynomial kernel captures the underlying patterns in the data accurately and provides robust classification results.

#### Radial kernel SVM (R-SVM)

In cases where prior knowledge about the data is lacking, the radial basis function (RBF) kernel is commonly employed to transform the data. The RBF kernel introduces two critical parameters, namely *C* and $\gamma$, which require careful consideration. The *C* parameter, commonly referred to as the regularization parameter, is shared among all SVM kernels and influences their behavior. A lower value for *C* promotes a smoother decision surface, while a higher value aims to classify all training sets accurately.

The $\gamma$ parameter, also known as the kernel coefficient, determines the influence of each training example on the decision boundary. Additionally, the $\sigma$ parameter, representing the standard deviation in the RBF kernel, controls the width of the kernel and influences the smoothness of the decision boundary. Choosing appropriate values for *C*, $\gamma$, and $\sigma$ is crucial, as they significantly impact the performance of the SVM model. It’s imperative to carefully tune these parameters to achieve optimal results.

The mathematical expression for the RBF kernel is as follows:

22 $$\begin{aligned} \ F(x_j, x_k) = \frac{1}{\sigma \sqrt{2\pi }} \exp \left( -\frac{1}{2}\left( \frac{x_j-x_k}{\sigma }\right) ^2 \right) \ \end{aligned}$$

This equation represents the transformed feature vectors obtained by computing the exponential of the squared difference between the input vectors $x_j$ and $x_k$ divided by the square of $\sigma$. The term $\frac{1}{\sigma \sqrt{2\pi }}$ serves as a normalization factor.

Choosing suitable values for the $C$, $\gamma$, and $\sigma$ parameters is critical in achieving optimal SVM performance. Careful parameter tuning enables the RBF kernel to capture complex relationships and non-linear patterns in the data, ultimately leading to improved classification results and better generalization.

## Results and discussion

The original spectrum DR8, as illustrated in Fig. 1, represents the unaltered measurements obtained without any modifications. This depiction offers an initial insight into the sample’s spectral profile, showcasing peaks, valleys, and patterns. However, it may incorporate undesirable baseline distortions or noise, potentially impacting accurate analysis and interpretation.

**Figure 4:**
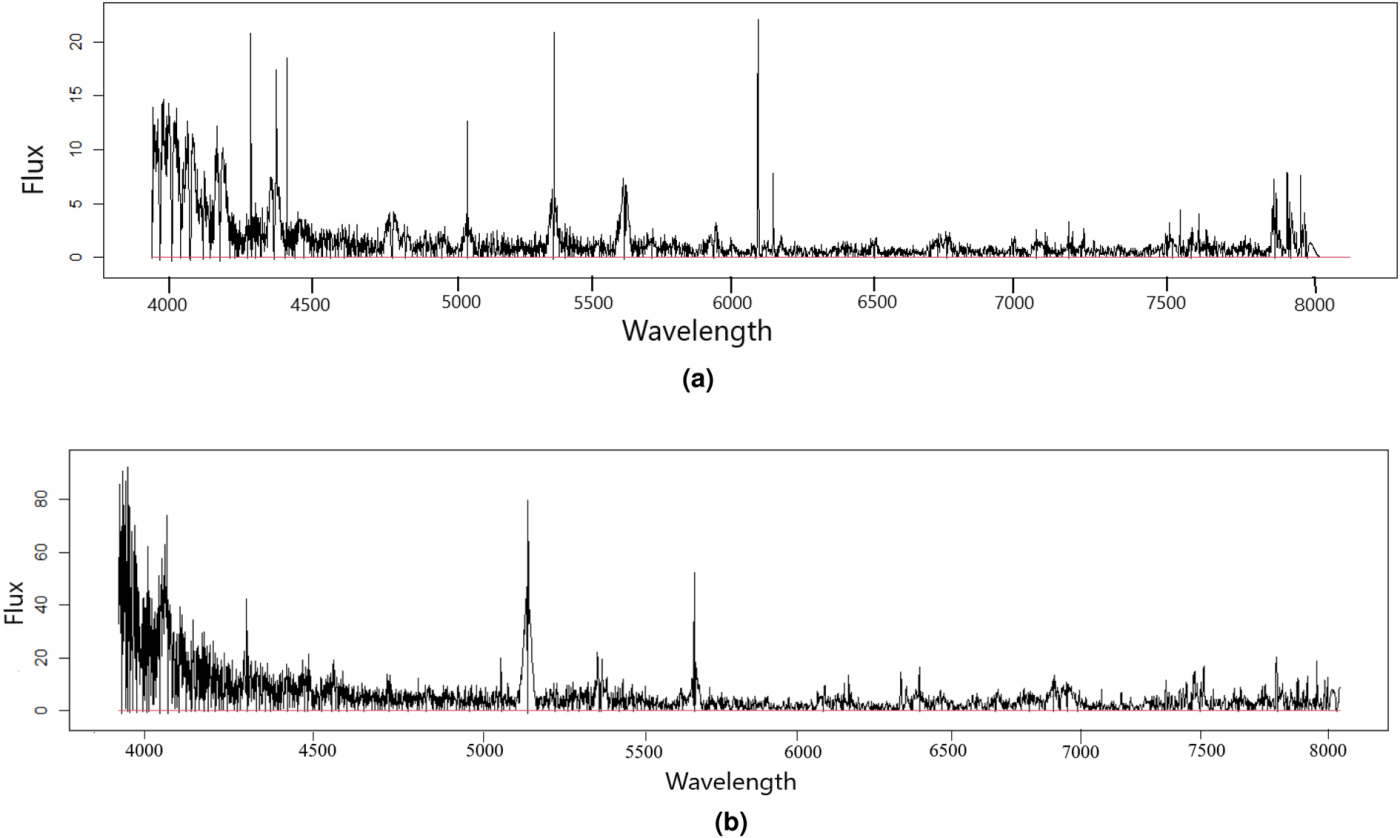
Displayed are the transformed spectrum and the baseline-corrected spectral for both types of stars. Baseline correction was conducted using the ALS method with a smoothing parameter of ($\lambda$) set to 2 and weights ($w$) at 0.001.

The figures displayed in Fig. 4 depict the transformed spectrum and baseline-corrected spectral data. The transformed spectrum, illustrated separately for both hot subdwarf stars (Fig. 4a) and B-type stars (Fig. 4b), showcases the data after undergoing a targeted transformation technique. This transformation is designed to amplify specific features, diminish noise, or accentuate particular regions of interest within the spectrum. Consequently, the transformed spectrum provides an alternative representation that may reveal hidden information or accentuate spectral traits that were less pronounced in the original data.

Moreover, the baseline-corrected spectral data displays in Fig. 4, the spectrum after undergoing baseline correction using the Asymmetric Least Squares (ALS) method, with a smoothing parameter ($\lambda$) set to 2 and weights (*w*) at 0.001. Baseline correction eliminates systematic variations or artifacts arising from uneven signal contributions or background interferences. Through the removal of baseline distortions, the baseline-corrected spectrum facilitates a more precise visualization and analysis of the spectral features and their relative intensities.

By comparing these three representations, researchers and analysts can gain valuable insights into the impact of transformation and baseline correction techniques on the spectral data, enabling more accurate interpretation and reliable analysis for their specific objectives.

To identify suitable training samples, the pan-core concept was utilized. This approach involved a two-step procedure.

Firstly, K-means clustering with a value of $K = 100$ was applied. This clustering algorithm grouped the data into 100 distinct clusters based on similarities in their features. Within each bunch, $s$ spectra were extracted using the nearest neighborhood method. This method aimed to select spectra closest to each cluster’s centroid, ensuring representative samples were chosen. As a result, 500 models (spectra) were obtained for both hot subdwarf and B-type stars.

These collected samples were then utilized to build support vector machine (SVM) models. Three SVM variants were trained: linear (L-SVM), polynomial (P-SVM), and radial basis (R-SVM). Each SVM variant had specific parameters that required tuning. For example, all SVM variants shared the ’cost’ parameter, representing the cost of misclassification and influencing the trade-off between achieving a wider margin and accurately classifying training data points. Additionally, the polynomial kernel required tuning the ’degree’ parameter, determining the polynomial degree of the kernel function.

**Figure 5:**
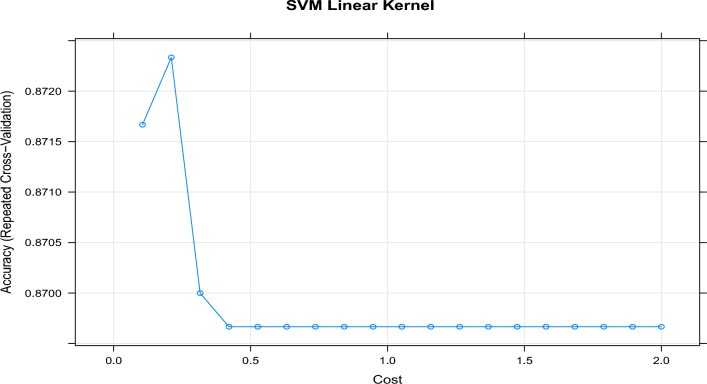
Tuning the cost parameter of a Linear Kernel Support Vector Machine (L-SVM) involves experimenting with various thresholds to optimize its performance.

**Figure 6:**
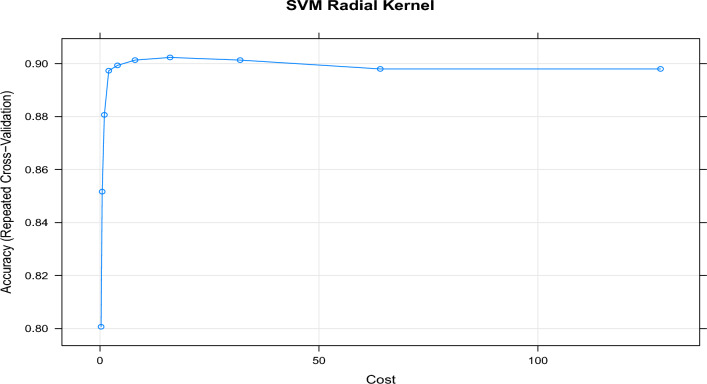
Tuning the cost parameter of a Radial Kernel Support Vector Machine (R-SVM) involves experimenting with various thresholds to optimize its performance.

To fine-tune the SVM models, repeated cross-validation was employed. This process involved assessing the cross-validated accuracy of the models using various values for the ’cost’ parameter. Figures 5 and 6 display the results of cross-validated accuracy for the SVM with the linear kernel and the SVM with the radial kernel, respectively. By utilizing the pan-core concept, extracting representative samples, and fine-tuning the SVM models through cross-validation, the approach aimed to enhance the accuracy and performance of the SVM models for classification or prediction tasks in the context of hot subdwarf and B-type stars.

The findings revealed that the SVM with a linear kernel achieved optimal performance with a regularization parameter ’cost’ set to 0.2, resulting in a repeated cross-validated accuracy of 87.2%. On the other hand, the SVM with a radial kernel achieved its best performance with a ’cost’ value of 21, resulting in a repeated cross-validated accuracy of 90.3%. These results underscore the significance of parameter tuning and offer insights into selecting the most suitable SVM model for accurately classifying B-type and hot subdwarf star spectra.

**Figure 7:**
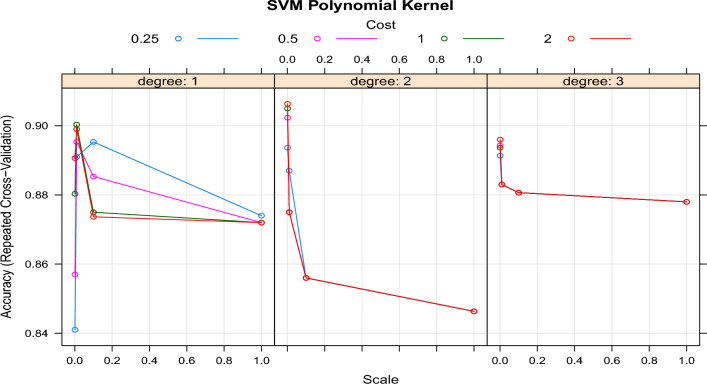
Tuning the cost and degree parameters of a Polynomial Kernel Support Vector Machine (P-SVM) involves experimenting with various thresholds to optimize its performance.

The cross-validated accuracy for the range of ’cost’ and ’degree’ parameters of the SVM with a polynomial kernel is illustrated in Fig. 7. The results indicate that the SVM with a polynomial kernel achieves optimal performance when the ’cost’ parameter is set to 0.001, and the ’degree’ parameter is set to 2. This combination results in a repeated cross-validated accuracy of 90.3%. These findings emphasize the importance of meticulously tuning the parameters to attain the best performance for the SVM with a polynomial kernel.

The accuracy of the models on the test data is also monitored. Table 1 presents the test and training accuracy, along with the optimal parameter(s), for all SVM models. This comprehensive evaluation allows us to assess the performance of each model and determine the effectiveness of the chosen parameters. By analyzing the results, we can gain insights into the generalization capability of the SVM models and make informed decisions regarding their deployment in practical applications.

**Table 1:** Comparative analysis of test and training accuracy with optimal parameter(s) for support vector machine (SVM) models across different evaluation metrics.

Model	Cost	Degree	Repeated cross-validation	Validation (test)	Calibration (training)
L-SVM	0.21	–	87.2	87.0	93.6
R-SVM	21	–	90.3	80.1	90.7
P-SVM	0.001	2.0	90.3	84.1	93.2

The results indicate that the SVM with a linear kernel achieves the highest accuracy of 87.0%, outperforming the SVM with a polynomial kernel (accuracy = 84.1%) and the SVM with a radial kernel (accuracy = 80.1%). This suggests that the linear kernel is better suited for the classification task in this study. The superior performance of L-SVM over P-SVM and R-SVM can be attributed to several factors. The L-SVM achieved the highest accuracy of 87.0%, surpassing the P-SVM’s 84.1% and the R-SVM’s 80.1%. This suggests that the data for B-type and hot subdwarf stars is likely more linearly separable in the feature space we used. The linear kernel’s simplicity helps prevent overfitting, which can be a risk with the polynomial and radial kernels, especially if the non-linear relationships in the data are not strong. Additionally, parameter tuning and cross-validation indicated that the linear kernel was more effective in generalizing to unseen data, further supporting the superior performance of L-SVM for this spectral classification task. These statistical results demonstrate the robustness and effectiveness of the linear SVM model in distinguishing between B-type and hot subdwarf stars.

The study holds significant value for astronomers, enabling them to classify stars based on their spectra with high accuracy, as demonstrated by the identification of 139 new hot subdwarf stars. By accurately categorizing stars, this research contributes to advancing and enriching astronomical knowledge. The discovery of these new stars underscores the efficacy of our approach in uncovering previously unidentified stellar objects with spectroscopic characteristics akin to hot subdwarfs. These findings not only enrich our understanding of stellar populations but also highlight the potential of our method to contribute to ongoing astronomical research endeavors aimed at cataloging and characterizing celestial objects.

## Conclusions

The analysis of the pan-core-based training data demonstrates the robust classification ability of the support vector machine (SVM) in distinguishing between hot subdwarf and B-type stars using their spectroscopic data. Remarkably, the average calibrated accuracy across all methods ranges from an impressive 90% to 94%. These results indicate the consistent and reliable performance of the SVM model in accurately categorizing the stars. Moreover, the validated accuracy of the SVM model, which assesses its effectiveness on previously unseen data, falls between 80.0% and 87.0%. This showcases the model’s ability to generalize and make accurate predictions beyond the training dataset. The SVM model employed a linear kernel with a carefully tuned cost parameter of 0.2, leading to an encouraging 87.0% correct classification rate for the spectrum data. This highlights the significance of parameter selection in optimizing the performance of the SVM model. The findings reaffirm the effectiveness of utilizing the SVM approach with pan-core training data, providing astronomers with a reliable and accurate method to differentiate stars based on their spectroscopic characteristics. Overall, this research contributes to the advancement of stellar classification and enhances our understanding of the distinguishing features of two kinds of stars. The reliable performance of the SVM model in this study demonstrates its potential as a valuable tool for astronomers, enabling more precise and efficient classification of stars based on their spectroscopic data. Additionally, our model’s success in identifying 139 new hot subdwarf stars highlights its broader impact in expanding our knowledge of stellar populations.

## Data Availability

The data utilized in this study are derived from the eighth and tenth data release (DR8 & DR10) of the LAMOST (Large Sky Area Multi-Object Fiber Spectroscopic Telescope) survey. The LAMOST DR8 and DR10 data can be accessed and downloaded from the official LAMOST data archive website (http://lamost.org). The data release includes a comprehensive catalog of observed objects, spectra, and associated metadata. To access the LAMOST data, researchers are required to register an account on the LAMOST data archive website and agree to abide by the data usage policy. Upon acceptance, users can access the data products and relevant documentation.
